# Psychological risks experienced by interpreters in the domestic violence cases: a systematic review

**DOI:** 10.3389/fsoc.2023.1139431

**Published:** 2023-08-17

**Authors:** Ning Guo, Olav Muurlink, Shane Doyle

**Affiliations:** School of Business and Law, Central Queensland University, Brisbane, QLD, Australia

**Keywords:** vicarious trauma, resilience, mental health, interpreting studies, police interviews, domestic violence

## Abstract

Interpreters occupy a complex position in police interviews involving domestic violence cases—neutral but necessary parties to traumatic content. The following systematic review explores the relatively sparse scholarly literature on interpreters' psychological responses to being a party to domestic violence interviews in a policing context. This article aims to explore themes of relevant studies targeting interpreters' mental health in such cases, with nine articles emerging from a comprehensive search of eight databases supplemented with a Google Scholar search. Various themes involving interpreters emerged from the ensuing analysis, including intrinsic difficulties, misguided expectations, role requirements, psychological impacts, posttraumatic growth, coping strategies, and recommendations for future research and practice, with findings holding implications for interpreting in other traumatic domains.

## 1. Introduction

International immigration has created increasingly multi-ethnic societies (Wallin and Ahlström, 2006), with new migrants globally experiencing cultural and language barriers that impede their communication in a range of contexts. The acculturation process of immigration, coupled with the lack of a common language, leaves immigrants needing regular access to interpreting services (Splevins et al., 2010). A particularly charged context for these encounters is domestic violence, with immigrants lacking fluency in the dominant language in highly multicultural societies such as Australia more likely to be victimized and particularly vulnerable due to their disconnection with extended family networks (InTouch, Multicultural Centre against Family Violence, 2014). Police typically engage interpreters to assist in investigative interviews with domestic violence victims, witnesses, and perpetrators (Martin and Valero Garcés, 2008), a role that invariably involves highly emotionally charged interview contexts.

Evidence suggests that interpreters experience an emotional toll from being party to these interviews. This emotional toll takes place against a background of considerable stress intrinsic to the interpreter's role. Moser-Mercer et al. (1998) found that interpreters' physiological and psychological responses seemed to be more negative due to task overload in contested settings. Sometimes, due to time constraints and the lack of help from organizations or peers, interpreting presents unpredictable risks, including incoherent information delivery and inappropriate interventions during interpretation (Giustini, 2021). Moreover, time pressure, the ill-defined role of interpreters, and the interpreting agencies' culture at times hinder investigators from properly briefing the interpreters regarding the investigation background and concerns (Walsh et al., 2020). Walsh et al. (2020) researched the role of interpreters during international criminal investigations and found that the ability of interpreters to substantially mitigate the emotional impact of such investigations was achievable in only a minority of cases. Darroch and Dempsey (2016) systematic review of sign language interpreters' experiences of transferred/vicarious trauma suggests that the task of the interpreter is intrinsically laden with emotional risk.

Research exploring the use of interpreters in forensic settings, including police-interpreter interviews and courtroom interviews, found a general lack of scrutiny of interpreter competence, including receiving inadequate training (Powell et al., 2017). Suggested shortcomings in the training of interpreters include a lack of instruction on dealing with the emotional challenges of their work. Various factors, including insufficient protective measures, interpreters' level of resilience, and lack of preparedness prior to the interpreting process, would all arguably take their toll on interpreters' psychological distress (McDowell et al., 2011; Crezee, 2015). In addition, numerous studies have indicated that community interpreters are at high risk of vicarious trauma and secondary traumatization while working with traumatized victims (see Lai et al., 2015; Mehus and Becher, 2016; Kindermann et al., 2017).

Interpreters have limited opportunities to receive education and training on gender-related violence (Norma and Garcia-Caro, 2016). In addition, scant scholarly attention has been paid to the emotional impacts of interpreter work, together with the extent to which trauma suffered by interpreters increased with greater exposure to victims of violence (Loutan et al., 1999). Also, the lack of scholarly inquiry focusing on the extent to which interpreters are impacted by the demands of their role can ultimately hamper their performance in the evidence-gathering process, together with potentially harming the victims' wellbeing (Powell et al., 2017).

### 1.1. Common terminology

This section will briefly explain common terminology used in describing interpreters' and other public service providers' psychological responses.

*Vicarious trauma* (VT) is a term often used to describe negative transformation in trauma workers' inner experience caused by empathetic engagement with traumatic content (Pearlman and Saakvitne, 1995), and will be the term preferred in this review. Other terms are more or less interchangeable with vicarious trauma, including “compassion fatigue,” “secondary traumatic stress” (Stamm, 1995, 1997), “secondary victimization” (Figley, 1995, 2002), and *secondary trauma* and *secondary traumatization* (ST; Pearlman and Saakvitne, 1995), all of which can be experienced by service workers engaged in frontline activities with traumatized clients (Diaconescu, 2015). Cieslak et al. (2014) utilized umbrella term *secondary traumatic stress* to discuss the impacts of secondary exposure as vicarious traumatization, compassion fatigue, and posttraumatic stress disorder (PTSD).

In contrast with vicarious trauma, there are a range of responses that do not rely on empathy as a mechanism of impact. *Compassion fatigue* can be understood as the cost of caring for vulnerable people (Figley, 1995), the emergence of a form of apathy as a result of the cumulative effect secondary trauma (Stamm, 2010). By contrast, Stamm (2010) described some positive and rewarding potential of working in traumatic interpreting settings as “*compassion satisfaction*,” defining it as the positive affect derived from performing valuable work (p. 8). Meichenbaum (1994) perceived some of these positive changes as post-traumatic growth engendered by witnessing others manage distress and thereby instilling hope to recover. Tedeschi and Calhoun (2004, p. 2) defined posttraumatic growth as “the experience of positive change that occurs as a result of the struggle with highly challenging life crises.” Specifically, self-perception, interpersonal relationships, and life philosophy are the three components of posttraumatic growth occurrence (Tedeschi and Calhoun, 1996; Manning et al., 2015).

Mathieu (2012) sees *burnout* as a term commonly used to describe professionals' physical and emotional fatigue when they lack job gratification and feel overwhelmed at work. Maslach et al. (2001) defined job burnout as a long-term three-dimensional response to job stressors: exhaustion, cynicism, and inefficacy. Burnout can lead to compassion fatigue, secondary traumatic stress, and secondary traumatization (Stamm, 2010) and is frequently linked to job turnover. Unlike other psychological syndromes, Diaconescu (2015) argues, burnout can be diminished by exposure to a supportive organizational climate.

In addition to the above terms, there are a range of terms that refer to ability to resist the emotional damage present in a traumatic environment. For example, emotional stress *resilience* is regarded as one of the non-cognitive elements of interpreters' protective toolkit (Chabasse, 2009).

### 1.2. Interpreting in police settings

As one aspect of community interpreting, legal interpreting is a broad field that incorporates court interpreting and interpreting for other legal processes or proceedings (Bancroft et al., 2013). There is a relatively broader scholarly inquiry into court interpreting than in non-courtroom contexts, such as policing settings, both in monolingual and bilingual situations (Eades, 2003).

An increasing number of law professionals and researchers acknowledge interpreters' assistance in negotiating the linguistic and cultural terrain of interviewing perpetrators and victims; their role is increasingly seen as vital in investigations when interviewing victims and witnesses (Milne and Bull, 1999). Legal interpreting is an extremely taxing professional area of expertise and tends to evolve into a separate area of specialization (Burley, 1990; Mayfield and Krouglov, 2019).

The involvement of interpreters in police interviews ranges from the *ad hoc* to highly structured and mandated. In Spanish police settings, the engagement of interpreters is often left to police officers' discretion; interpreters are assigned various tasks, including assisting police in performing an arrest, translating related documents, and questioning involved parties (Martin and Valero Garcés, 2008). By contrast, in Australia, the use of interpreter in police operations is covered in police standing orders (Mulayim et al., 2014). Compared to policing contexts, more research targeting court interpreting has been undertaken, both in monolingual and bilingual situations (Eades, 2003). This is partly because court interpreting is more often conducted in the public eye, while police interviews using interpreters are more often conducted in relative obscurity (Mulayim et al., 2014). Researchers, including Gallai (2013), highlighted this gap in the literature and addressed the need for further research, particularly the imperative to enhance public scrutiny when interpreters assist police during investigative interviews.

While certified interpreters are always considered the first option when working in Australian police systems (Lai, 2016) most Australian interpreters were trained on a general basis, with no specialist training regarding police interpreting (Lai, 2016). Despite most interpreters' exposure in contesting contexts and the complexity of the job, the interpreter training in Australia lacks breadth (Guo et al., 2023). While interpreters are needed in most police interviews, many police in Australia still concern about the job performance of police interpreters, with potential bias if interpreters could take sides (Goodman-Delahunty and Howes, 2019; Hale et al., 2019).

### 1.3. Taking cultural context into account

Interpreters are required to convey information in an emotionally and culturally congruent way (Prentice et al., 2014)—a complex taxing task. Extra literature supplemented that it is noteworthy that interpreters who share the same language with clients may not necessarily share the same culture (Berthold and Fischman, 2014), despite assumptions held by other involved parties. In practice, the interpreters' cultural background will often not perfectly match their clients; different cultural backgrounds between interpreters and clients may subsequently trigger potential bias (Engstrom et al., 2010; Berthold and Fischman, 2014). Cultural issues relating to sensitive topics, especially gender-based crime, can have a dramatic impact on the interpreter's performance (Powell et al., 2017; Walsh et al., 2020).

### 1.4. The interpreter's role

When conveying emotional and cultural information, the act of interpreting ideally demands that the interpreter adopts a “backseat,” neutral, unobtrusive role (McDowell et al., 2011). Interpreters can shift into the role of clients' advocates when processing their verbal and non-verbal information (McDowell et al., 2011; Prentice et al., 2014). In addition, in the seemingly simple act of information transfer, interpreters perform multiple functions, including catalysts, cultural counselors, and clients' advocates (Prentice et al., 2014). Interpreters can shift into the role of clients' advocates when processing their verbal and non-verbal information (Prentice et al., 2014).

### 1.5. Impacts on interpreters and coping mechanisms

According to McDowell et al. (2011), interpreting is physically and mentally taxing, requiring competency in language skills, knowledge of cultural customs, and interpersonal relationship skills, and the “reading” of non-verbal cues. Against this background of inherent complexity, Tipton (2017) noted that relevant stakeholders highlighted a lack of understanding about the emotional toll of the work. Research by Mirdal et al. (2012) on therapists and interpreters in professional psychology settings revealed a link between compassion, vicarious victimization, and stress-related burnout in line with the findings of with Darroch and Dempsey (2016) review. Based on Harvey (2001) research targeting psychologists' perspectives on interpreters, interpreters who work alongside mental health professionals might be equally vulnerable to occupational stress but lack adequate training to mitigate these adverse impacts.

The broader literature on coping strategies in the context of professional interpreting is also evolving rapidly. Holmgren et al. (2003), for example, found that some refugee interpreters detached themselves from traumatic contents and applied self-control coping strategy to stabilize themselves. Some interpreters avoid emotionally taxing interpreting activities to escape being exposed to trauma. However, avoidance is never an effective long-term strategy when dealing with trauma (Brewin and Holmes, 2003).

Catherall (1995) research on therapists took a less avoidant approach, emphasizing the potential benefits of peer groups, particularly in alleviating emotional disturbance and detecting and clarifying distorted viewpoints resulting from close exposure to traumatic work environments. Similarly, Mahyub-Rayaa and Baya-Essayahi (2021) point to peer support and professional psychological consultation as potentially effective in building resilience in alleviating the emotional burden of interpreting (Anderson, 2011). Moreover, organizational support can ensure interpreters access peer-group debriefing as a means of tackling emotional stress (Anderson, 2011). Strategies such as the provision of pre-meetings and engaging in other preparatory activities (Powell et al., 2017) have also been raised as potential responses.

Finally, the promulgation of appropriate policies and practices concerning the use of interpreters is also recommended for professional or humanitarian organizations (Valero-Garcés, 2005). Despite this body of work, much of it theoretical rather than based on primary research, enhancing interpreters' working conditions and improving the status/understanding of roles performed by professional parties in emotionally challenging contexts warrants further exploratory research (Holmgren et al., 2003).

## 2. Study aims

This systematic review aims to synthesize the available peer-reviewed scholarly evidence, exploring the emotional experiences of interpreters in a particularly challenging context: during the police interview process involving cases of domestic violence. It builds on the work by Darroch and Dempsey (2016) who conducted a review looking at the emotional challenges faced by sign language interpreters. The following review question was developed using the PICO (Population, Intervention, Compassion, and Outcome) framework (Tawfik et al., 2019) and asked, “*what are the psychological effects on interpreters when working with police involving cases of domestic violence?*”

### 2.1. Inclusion criteria

Drawing upon the review protocol of Stern et al. (2014), this study focuses on scholarly papers that meet the following inclusion criteria: (i) journal articles published since 2000; (ii) written in English; (iii) with full-text available; and (iv) presenting empirical research, as opposed to purely theories, concepts or literature reviews. Papers that did not include an explicit focus on emotional or psychological impact were excluded, as well as papers that included interpreting/translation but not in a professional policing context. For example, various papers that referred to bilingual family members or bystanders involved in interpreting within domestic violence contexts were excluded from the study.

### 2.2. Search strategy

In 2021, the following eight electronic databases were utilized in the search, owing to their relevance to the social sciences: Wiley Online, Taylor and Francis Online, Web of Science, Scopus, Sage Journals, CINAHL, Embase, and PsycINFO. The search strategy involved directly searching the databases using the search strategy below, followed by a search of the respective reference lists. Consistent with the method recommended by Butler et al. (2016), each database was interrogated by the researcher in consultation with an expert librarian, and each term was truncated where appropriate, combined with the Boolean operator “AND” or “OR.” In addition, a supplementary search was conducted in Google Scholar to ensure that the inclusion of all pertinent literature to the best extent possible.

A full list of search terms can be found in Table 1. An initial search was conducted using the terms (interpreter^*^ OR interpreting) AND (“domestic violence”) AND (“police interview^*^”), which failed to retrieve any relevant results. Subsequently, a broader search was undertaken by removing search terms involving either domestic violence or police interviews. The second round of search terms combined “interpreter^*^” OR “interpreting” with “domestic violence,” “domestic abuse,” “domestic violence and abuse,” “gender violence,” “GV, “DV,” “DVA,” “police,” and “police interview^*^.”

**Table 1:** Search terms.

**Databases**	**Search terms**		**Total**
	“interpreter^*^” AND “domestic violence” “interpreter^*^” AND “DV” “interpreter^*^” AND “domestic abuse” “interpreter^*^” AND “domestic violence and abuse” “interpreter^*^” AND “DVA” “interpreter^*^” AND “family violence” “interpreter^*^” AND “gender violence” “interpreter^*^” AND “sexual violence” “interpreter^*^” AND “GV” “interpreter^*^” AND “intimate partner violence” “interpreter^*^” AND “police” “interpreter^*^” AND “police interview^*^”	“interpreting” AND “domestic violence” “interpreting” AND “DV” “interpreting” AND “domestic abuse” “interpreting” AND “domestic violence and abuse” “interpreting” AND “DVA” “interpreting^*^” AND “family violence” “interpreting” AND “gender violence” “interpreting^*^” AND “sexual violence” “interpreting” AND “GV” “interpreting” AND “intimate partner violence” “interpreting” AND “police” “interpreting” AND “police interview^*^”	
Embase	7	44	51
Wiley	10	57	67
Taylor and Francis	12	41	53
Scopus	83	195	278
Web of Science	79	182	261
Sage	3	15	18
CINAHL	5	9	14
PsycINFO	4	3	7
Manual search	**218**		
Total	**967**		

### 2.3. Study selection

The review process used two independent reviewers—the first and second authors—using Rayyan software, which allows reviewers to examine papers for inclusion independently, blind to the other's choice, and then reconcile differences. Differences between the reviewers were settled through discussion and more detailed reading. Finally, included articles were downloaded for full-text reading.

**Figure 1:**
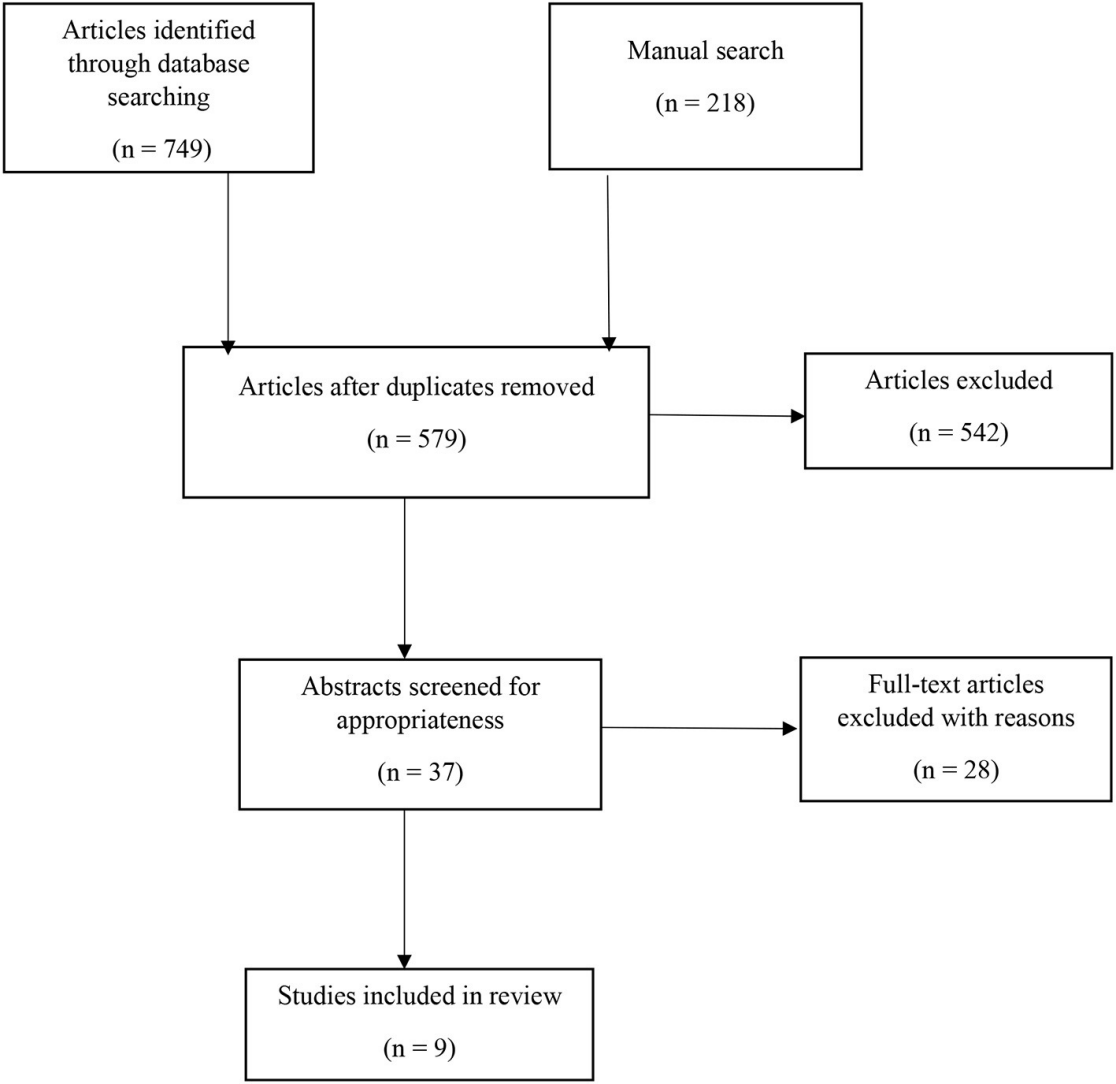
Flow chart.

The preliminary search identified 967 relevant studies. All studies were saved using Endnote and screened for duplicates. Following the removal of duplicates, 579 journal articles remained. The systematic search yielded a limited number of results specifically focused on domestic violence studies. Therefore, research pertaining to interpreters in the context of domestic violence was included as eligible consideration. After two rounds of the screening process by reading the title, abstracts, and keywords, 37 articles were regarded as eligible articles, which were subsequently printed in hard copy for further reading and assessment. However, only nine studies that met inclusion criteria (see Section 2.1 above) were finally included in the review. A list of all nine remaining articles can be found in Appendix A. Figure 1 provides a flowchart of the literature search strategy.

A data extraction form based on Preferred Reporting Items for Systematic reviews and Meta-Analysis for Protocol (PRISMA, Tawfik et al., 2019) and the quality assessment form titled the Mixed Methods Appraisal Tool (MMAT; Hong et al., 2018) was also applied to assess the articles' quality.

### 2.4. Overview of papers

A total of nine studies remained after the search process was concluded, which were then then categorized using Table 2. The heterogeneity of interpreting services was not thoroughly examined by included studies. Most studies were directed at discussing the experiences and perspectives of the role of interpreters working with different professionals, including two studies that explored the challenges of using interpreters drawn from the perspectives of professionals. The remaining studies explored various issues, including emotional stress and coping strategies that emerged during the interpretation process.

**Table 2:** Overview of included papers.

**References**	**Locations**	**Research design**	**Data collection**	**Research approach**	**Key findings**
Del Pozo-Triviño and Toledano-Buendía (2017)	Spain	Mixed methods	Focus groups; Questionnaire survey; Semi-structured interviews	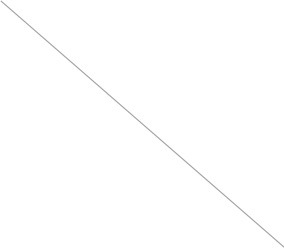	The paper defines the characteristics of police and court interpreting for victims of gender violence and ascertains the training needs of interpreters in these contexts. Specialized training is needed in building communication bridges between victims and service providers.
Doherty et al. (2010)	UK	Quantitative	Surveys and questionnaires	Grounded theory	The study highlights the need for good practice guidelines to support interpreters in mentally demanding work. Interpreters in this study experienced a range of emotions related to their work, including anger, sadness, hopelessness, and powerlessness. Posttraumatic growth also exists alongside interpreting.
Engstrom et al. (2010)	USA	Qualitative	In-depth interviews	Ethnography	The study highlights the need for proper training of interpreters and preventing against their exposure to vicarious trauma.
Kindermann et al. (2017)	Germany	Quantitative	Cross-sectional survey and questionnaire	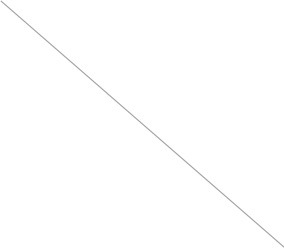	Significant correlations emerged between primary and secondary traumatization among interpreters, which is partially mediated by low secure attachment.
Lai and Costello (2021)	AU	Qualitative	Focus groups	Phenomenology	Interpreters in Australia are at risk of experiencing vicarious trauma due to working in public service settings. Vicarious trauma can take a significant toll on the mental health and wellbeing of interpreters, and their ability to perform job effectively.
Lai et al. (2015)	AU	Quantitative	Online survey	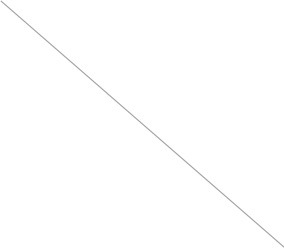	Public service interpreters in Australia are exposed to traumatic client material, which can significantly affect their cognitive processes and emotions during and after the job. Interpreters use a range of coping strategies to manage the impact of traumatizing material, including seeking support from colleagues and mental health professionals. The study highlights the need for institutional care and self-care for to interpreters exposed to traumatic material.
Mayfield and Krouglov (2019)	UK	Quantitative	Questionnaires	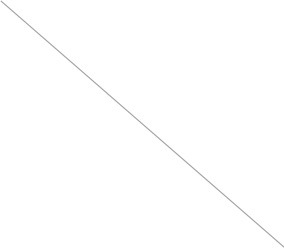	The study identified a range of inconsistencies, issues, and challenges interpreters face in facilitating communication with non-English speaking victims and witnesses during police investigations and statement taking procedures.
Powell et al. (2017)	AU	Qualitative	Interviews	Thematic analysis	The interpreters' lack of preparedness to deal with the traumatic and sensitive nature of children's abuse histories is a major challenge in using interpreters in child sexual abuse interviews. An insufficient understanding of the optimal child interview process is another challenge in using interpreters in child sexual abuse interviews.
Splevins et al. (2010)	UK	Qualitative	Interviews	Phenomenology	The study explored the vicarious experiences of interpreters working with asylum seekers and refugees in therapeutic settings. Although all participants experienced distress, they also perceived themselves to have experienced posttraumatic growth in some way.

All selected studies were conducted in “developed countries,” which may be attributed (in part) to confining studies only published in English. One study was conducted in the United States, two in continental Europe, with the balance in the United Kingdom (three) and Australia (three).

One study employed mixed methods combining focus groups, survey, and interviews. Four studies employed a qualitative approach, chiefly using interviews (one employed a focus group approach). The remaining four studies deployed a quantitative approach that was equally represented in surveys and questionnaires (one each) and a combination of surveys and questionnaires (two each).

## 3. Key themes emerging from this review

Eight key themes relating to the psychological challenges faced by interpreters emerged from the analysis and were categorized as: 1. “Intrinsic difficulties,” 2. “Misguided stakeholder expectations,” 3. “Mismatch in cultural contexts,” 4. “Multiple roles,” 5. “Impact pathways,” 6. “Training/support issues,” 7. “Post-traumatic growth,” and 8. “Coping strategies and recommendations.” Table 3 presents the themes and the papers they emerged in. Note that in some cases these themes emerged not from primary evidence, but in the authors' analysis/discussion.

**Table 3:** Key themes.

**Themes**	**General notions from studies**	**References**
Intrinsic difficulties	Physically and mentally involved work Miscommunication	Engstrom et al., 2010; Lai et al., 2015; Mayfield and Krouglov, 2019
Misguided stakeholder expectations	Unsatisfaction from other parties Other parties lack collaboration and understanding with interpreters	Mayfield and Krouglov, 2019; Lai and Costello, 2021
Mismatch in cultural contexts	Interpreters' cultural backgrounds do not always match up with clients Difficulties dealing with cultural differences including cultural taboos	Engstrom et al., 2010; Splevins et al., 2010; Powell et al., 2017; Mayfield and Krouglov, 2019; Lai and Costello, 2021
Multiple roles	Invisible person Cultural broker Impartial helper Facilitator	Splevins et al., 2010; Del Pozo-Triviño and Toledano-Buendía, 2017; Powell et al., 2017; Mayfield and Krouglov, 2019; Lai and Costello, 2021
Impact pathways	Empathy/identification with clients Physical uncomfortableness Emotional distress Over-empathy Depression and anxiety Affecting personal life Vicarious trauma Secondary traumatization	Doherty et al., 2010; Engstrom et al., 2010; Splevins et al., 2010; Lai et al., 2015; Kindermann et al., 2017; Powell et al., 2017; Lai and Costello, 2021
Training/support issues	Lack of preparedness and understanding of the optimal practice Lack of specific theme training and well-established training protocol Lack of status recognition and respect Lack of professional support	Engstrom et al., 2010; Splevins et al., 2010; Lai et al., 2015; Del Pozo-Triviño and Toledano-Buendía, 2017; Powell et al., 2017; Mayfield and Krouglov, 2019; Lai and Costello, 2021
Posttraumatic growth	Personal growth (relations with other parties, sense of achievement, skill upgradation, learning different cultures, and deepening sense of compassion)	Doherty et al., 2010; Splevins et al., 2010
Coping strategies and recommendations	Avoidant coping Debriefing Detachment and self-control Social support Self-care activities Preparatory communication with other professionals Further education, specialization, and refinement of skills Feedback mechanism for negative experiences	Doherty et al., 2010; Engstrom et al., 2010; Splevins et al., 2010; Lai et al., 2015; Del Pozo-Triviño and Toledano-Buendía, 2017; Kindermann et al., 2017; Powell et al., 2017; Mayfield and Krouglov, 2019; Lai and Costello, 2021

### 3.1. Intrinsic difficulties

Mayfield and Krouglov (2019) found that performing interpreting services for domestic violence victims and witnesses was more challenging than (for example) interpreting in the interrogation of suspects. Interpreters may struggle with conveying linguistic content and cultural context of the story accurately (Engstrom et al., 2010). As a result, inaccurate and inconsistent interpretation can emerge, with interpreters needing to avoid confabulation in cases of nuanced information (Engstrom et al., 2010).

### 3.2. Misguided stakeholder expectations

With interpreters working in domestic violence interviews, police officers may attempt to shift responsibilities outside the purely interpreting duties (Mayfield and Krouglov, 2019). With the power imbalance between police and interpreters in this setting, interpreters can feel they are being coerced by police (Mayfield and Krouglov, 2019). Lai and Costello (2021) discovered that there might be some lack of recognition among stakeholders about the taxing nature of interpreting work and the need to recognize the impact on interpreters' mental wellbeing.

### 3.3. Mismatch in cultural contexts

A number of studies suggest that interpreters' background characteristics can influence the interview outcome and consequently impact the validity of the interview, a finding consistent with Jentsch (1998) earlier research. On the one hand, shared commonalities between the interpreter and the client, such as religious beliefs, gender, cultural background, and social class, can act as the catalyst for establishing rapport with one another (Lai and Costello, 2021) but at the same time, this rapport can lead to premature assumptions on the part of the interpreter or the domestic violence victims. Splevins et al. (2010) found that when there was a shared cultural background, interpreters' emotional mirroring with clients was also more common.

On the other hand, non-matching religious beliefs, ethnicities, or political conflicts between interpreter and victim may impede information disclosure and distort the interview content (Engstrom et al., 2010). At times interpreters need to overcome, or be aware of, cultural taboos that may hinder the quality of communication (Mayfield and Krouglov, 2019). Powell et al. (2017) argued that cultural taboos might compromise interpreters' professional performance, especially cultural taboos involving gender issues. Some interpreters may face barriers to help-seeking due to cultural stigma, such as mental illness, and it should be left up to relevant stakeholders to create a supportive work environment to acknowledge the cultural diversity in workplace (Lai and Costello, 2021). Even though language does not equate to culture, not surprisingly, interpreters are required to undertake the role of “cultural broker,” to facilitate cross-cultural understanding (Splevins et al., 2010).

### 3.4. Multiple roles

Lai and Costello (2021) described how stakeholders, including police, regarded interpreters as marginal importance to the encounter, but in fact more commonly interpreters are playing active and multiple roles. In Del Pozo-Triviño and Toledano-Buendía (2017) research, interpreters played the role of building communication bridges between the public services and victims, or of cultural “broker” (Splevins et al., 2010). In Mayfield and Krouglov (2019) study, the majority of police interpreters regarded their role as impartial helpers, with a small percentage viewing them as facilitating the whole communication exchange, a role that Powell et al. (2017) also found in their study of interpreters working with child complainants.

### 3.5. Impact pathways

A number of papers included in this review highlight that interpreting can have considerable negative psychological impacts on interpreters, despite what can be seen as a technical and unobtrusive role. For instance, the very act of being consigned to the background during interviews can place considerable demands on interpreters, including a negative emotional toll (Powell et al., 2017). Interpreters who are not adequately prepared to deal with the traumatic and sensitive nature of abuse can be emotionally affected by the traumatic contents, which could further affect victims' willingness to disclose further information (Powell et al., 2017). The unobtrusive role demands can present various emotional effects on interpreters, including helplessness, isolation, anxiety, depression, indifference, and sadness (Doherty et al., 2010; Lai et al., 2015). When dealing with traumatic cases, interpreters frequently experience strong empathetic feelings toward their clients and feel “torn” emotionally by conflict between their role and the content of interviews (Doherty et al., 2010; Engstrom et al., 2010). Furthermore, Doherty et al. (2010) suggested that nearly one third of respondents reported having difficulty moving onto next job due to the aftereffects of distress from a previous interpreting job.

In line with research on interpreters working in other stressful settings, interpreters working with domestic violence victims may experience vicarious trauma or defensive reactions designed to distance themselves from the emotional impact of being exposed to the content of interviews (Splevins et al., 2010). Lai et al. (2015) found that about four in five interpreters reported experiencing distress caused by exposure to traumatic client content, including domestic violence settings. When interpreting a victims' harrowing experiences, whether the interpreters have experienced a similar situation or not, they are still prone to be affected (Lai and Costello, 2021). Interpreters may experience burnout, compassion fatigue, and other forms of psychological distress because of their demanding work (Lai and Costello, 2021). Kindermann et al. (2017) in their research on domestic violence in a refugee setting found that interpreters who had a prior history of trauma and those with a lack of social support and low sense of coherence were particularly vulnerable to secondary traumatization.

### 3.6. Training/support issues

Considering the evidence of emotional impact, the literature suggests interpreters lack formal skills to work effectively in delicate or distressing contexts. For instance, Powell et al. (2017) examined sexual abuse investigations and found that interpreters were underprepared for such cases. The lack of specialist training in specific contexts, subsequently undermined their performance in effectively undertaking their role (Mayfield and Krouglov, 2019). American researchers Engstrom et al. (2010) mentioned that this situation is compounded by the lack of accredited training on offer to interpreters involving less directly trauma-related skills such as ethics, note-taking techniques, and mastery of technical terminology. Engstrom et al.'s team add that, there is no well-established protocol for training professional interpreters in community settings due to the disparity in guidelines for interpreters and the lack of standard qualifications to become eligible interpreters (Engstrom et al., 2010).

The literature as a whole thus supports the need for improvements in the quality of interpreter training beyond current practice, particularly to incorporated psychological skills (Del Pozo-Triviño and Toledano-Buendía, 2017). This is related to the question of how to enhance the occupational status of interpreters and raises the broader question of interpreting as a profession, let alone interpreting in trauma settings as a specialization. Lai and Costello (2021) pointed out that national bodies in some cases set minimum standards for interpreter training and credentialing, but those standards may not be sufficient to prepare interpreters for emotional demands of their work.

The provision of training is particularly problematic, because a substantial proportion of interpreters are freelancers engaged on a casual basis, despite being employed indirectly by recruitment agencies (Splevins et al., 2010), so they tend to lack proper organizational support when an emergency occurs. Study by Lai et al. (2015) found that only one in five interpreters sought support from counselors, and only 14% from therapists. Therefore, interpreters' lack organizational support could exacerbate their mental issues.

### 3.7. Posttraumatic growth

While most of the literature points to stress and vicarious trauma as a result of involvement in domestic violence interviews, the experience need not be uniformly negative. Splevins et al. (2010) found that over time, the negative emotions dissipated, and positive emotions predominated. Two studies highlighted interpreters' positive emotional reactions when interpreting, including describing the role as gratifying, stimulating, and helpful (Doherty et al., 2010; Splevins et al., 2010). Splevins et al. (2010) also found that interpreters articulated more cohesive relationships with their clients and service providers and felt more compassionate and altruistic in their work. Interpreters' perceived changes in self and life philosophy can be inferred that interpreters may find their work rewarding in terms of personal growth and development (Splevins et al., 2010). These researchers also found that interpreters sensed feeling valued for providing a service that helped others. Some interpreters also described a diverse array of rewarding aspects associated with their work, including expanding their knowledge base, skills enhancement, and receiving positive feedback and recognition from participating parties (Doherty et al., 2010).

### 3.8. Coping strategies and recommendations

A review of the scholarly evidence has revealed that interpreters employ various coping mechanisms to deal with the demands of their occupation. Not all coping mechanisms are adaptive, however. Interpreters for example, in some cases simply avoid emotionally demanding jobs that might negatively impact their emotional stability (Doherty et al., 2010), or actively practice emotional detachment a (Lai and Costello, 2021). More positively, interpreters seek support from colleagues, family, friend, counselors, and therapists (Lai et al., 2015; Lai and Costello, 2021).

Splevins et al. (2010) and Doherty et al. (2010) concluded that interpreters developed strategies ranging from exercising, watching films, mediating, and turning to religion to deliberately avoidant coping techniques. Also, interpreters learned coping on the job or through acting as bystanders to the process, which allowed them better coping with their own distress (Splevins et al., 2010).

The provision of pre-meetings and engaging in other preparatory activities beforehand is raised in the literature as one pathway to improving interpreters' work efficiency and accuracy (Powell et al., 2017) and thus reduce the intrinsic difficulty of the job. Enhanced self-care, including regular work breaks and exercise, can also alleviate their mental distress (Doherty et al., 2010). Again, as noted in the previous section, there is a role for training. Researchers advocated more specialized training and screening to prepare interpreters for the emotional demands of interpreting in traumatic police interviews (Doherty et al., 2010; Engstrom et al., 2010; Splevins et al., 2010; Del Pozo-Triviño and Toledano-Buendía, 2017; Kindermann et al., 2017; Powell et al., 2017; Mayfield and Krouglov, 2019).

Doherty et al. (2010) found that interpreters view improvements in training and the level of interpreter support as being important factors in helping them better manage the demands of interpreting. Powell et al. (2017) argue that interpreters should be trained to exhibit a professional demeanor, engender mutual trust with clients, and help facilitate clients' disclosure. In addition, Lai et al. (2015) suggested that interpreter training programs should include curricula on trauma and its effects and organizations should have policies in place to support those who may become traumatized in workplace.

Finally, there appears to be a need for support services and supervision to help interpreters care for themselves. Lai and Costello (2021) mentioned the need for interpreting agencies to foster a sense of trust and support for interpreters, instead of creating a culture of fear and uncertainty when disclosing distress form work. Furthermore, the establishment of a feedback mechanism for interpreters to provide feedback about their negative experiences with others may also help better tackle trauma (Lai and Costello, 2021).

## 4. Discussion

Interpreters play a complex and demanding role in police interview/domestic violence settings that require (amongst other things) mental attentiveness, processing information, and conveying clients' emotions. This review indicates that in addition to these technical requirements, interpreters face considerable psychological challenges in completing their duties without sustaining emotional damage. The scenario also requires the interpreter to be both highly attuned to the context, but at the same time retain professional and emotional separation. Despite the complexity of roles such as this, the field of interpreting is still not commonly recognized as a profession as this vocation lacks official recognition, a situation compounded by generally accepted standards that vary worldwide (Rajpoot et al., 2020).

The limited literature available focuses on stressors impacting professionals, more broadly, and highlights that researchers hold divergent opinions regarding the risk factors causing professionals' stress. In fact, researchers such as Devilly et al. (2009) asserts that mental health professionals' exposure to clients' traumatic material had no significant impact on burnout, vicarious trauma, or secondary traumatic stress for service providers like mental health professionals. These researchers instead argued that the stress involved with the work itself, such as being new to the vocation, caused professionals the most distress. Findings from this literature review are at odds with this viewpoint; the small body of literature generally accepts the view that interpreters are at high risk of vicarious victimization and stress-related burnout (Lai et al., 2015; Lai and Costello, 2021). However, less attention has been focused on the potentially negative psychological impacts on service providers exposed to the traumatic experiences of victims (McCann and Pearlman, 1990). Working with victims may contribute to interpreters' feeling helpless and hopeless (McCann and Pearlman, 1990. Furthermore, they may find their cognitive processes disrupted due to long-term exposure to traumatic information in the workplace (Paivio, 1990).

In response to these challenges, there appears to be a clear need for ongoing training to assist interpreters in more effectively coping with emotional challenges associated with the interpreting process (Valero-Garcés, 2005; Del Pozo-Triviño and Toledano-Buendía, 2017). However, there is no set standard or protocol which addresses minimum training requirements for interpreters' mental wellbeing. For example, in Australia, National Accreditation Authority for Translators Interpreters (2016) does not require any wellbeing competency in its interpreter attributes. This is at odds with view such as Pochhacker (2015) who argues that stress resistance and stamina (trainable skills) are pivotal for aspiring interpreters, and Oraki and Tajvidi (2020, p. 52) who identified that the occupational requirements of interpreting could be clustered under two main competencies: common and specific, amongst which stress management was listed as a competency that can be developed. It is recommended that training institutions including universities should consider the critical importance of including curriculum on wellbeing. While no doubt, as some scholars argue, both interpreters and investigators must bear some responsibility for being as well prepared as possible prior to commencing the interview process (Holmgren et al., 2003; Miller et al., 2005; Prentice et al., 2014), the profession as it stands seems relatively ill prepared.

## 5. Limitations

The power of this review is clearly by the fact that the literature is quite sparse. It is possible that by extending the search terms and including the gray literature, the body of relevant work could be expanded. It is also possible that unpublished police reports and papers may address some of the issues raised—in the field, together with the likelihood that studies exist in languages, other than English, that address this field. Besides, the heterogeneity of interpreting services was not explored and required further exploration. Interpreters' variations may influence their emotional stability and the generalizability of the research findings, which were not discussed in this review either.

## 6. Conclusion

Despite lacking the formal status of a profession, interpreting is a complex and demanding undertaking, requiring interpreters to respond to clients, often within emotionally charged and stressful contexts, in an unobtrusive and reserved manner. The extent to which interpreters are psychologically impacted by their role is inextricably intertwined with interpreters' mental wellbeing, the neutrality of involved parties, and the collaboration with service providers like police officers during investigative interviews.

Nonetheless, empirical research exploring the psychological impacts experienced by interpreters working in police settings is scarce, particularly when dealing with domestic violence situations, the focus of this review. Through a comprehensive search of eight databases and one search engine, this study revealed that the risks of vicarious trauma occurring in the context of interpreting are likely to be pronounced, particularly within traumatic contexts. Instances where interpreters are required to work with police in domestic violence cases are not uncommon, and evidence suggests that interpreting cases involving trauma can seriously impact their career and personal life, both in a positive and negative manner. Therefore, there is a need for further research on interpreting in potentially traumatizing contexts such as domestic violence investigations.

In summary, despite a relatively small body of existing literature, there already appears to be a clear consensus on the need to broaden the scope of training for interpreters to better equip themselves to deal with the role demands posed by interviews in these demanding and stressful contexts. This research highlighted that further research is needed regarding interpreter training, including building resilience, developing interpreters' cultural competence and domain-specific training in domestic violence and police interviews.

## Author contributions

NG and OM contributed conception and design of the study. NG performed database searches and statistical analysis in agreement with OM and SD. NG wrote the first draft of the manuscript. All authors contributed to the manuscript revision and read and approved the submitted version.
