# Supplementary material for: Psychological risks experienced by interpreters in the domestic violence cases: a systematic review
Source: Front Sociol. 2023 Aug 17;8:1139431. doi: 10.3389/fsoc.2023.1139431 (PMC10469901; doi:10.3389/fsoc.2023.1139431)
Supplement: Supplementary file 1 [file Table_1.docx]

**Appendix A: Included Studies**

| **Included 9 studies** |
| --- |
| Del Pozo-Triviño, M., & Toledano-Buendía, C. (2017). Training interpreters to work with foreign gender violence victims in police and court settings. *Language and Law= Linguagem e Direito, 3*(2). |
| Doherty, S. M., MacIntyre, A. M., & Wyne, T. (2010). How does it feel for you? The emotional impact and specific challenges of mental health interpreting. *The Mental Health Review, 15*(3), 31. <https://doi.org/10.5042/mhrj.2010.0657> |
| Engstrom, D. W., Roth, T., & Hollis, J. (2010). The use of interpreters by torture treatment providers. *Journal of Ethnic & Cultural Diversity in Social Work, 19*(1), 54-72. <https://doi.org/10.1080/15313200903547749> |
| Kindermann, D., Schmid, C., Derreza-Greeven, C., Huhn, D., Kohl, R. M., Junne, F., ... & Nikendei, C. (2017). Prevalence of and risk factors for secondary traumatisation in interpreters for refugees: a cross-sectional study. *Psychopathology, 50*(4), 262-272. <https://doi.org/10.1159/000477670> |
| Lai, M., & Costello, S. (2021). Professional Interpreters and Vicarious Trauma: An Australian Perspective. *Qualitative Health Research, 31*(1), 70-85. <https://doi.org/10.1177/1049732320951962> |
| Lai, M., Heydon, G., & Mulayim, S. (2015). Vicarious trauma among interpreters. *International Journal of Interpreter Education, 7*(1), 3-22. |
| Mayfield, K., & Krouglov, A. (2019). Some aspects of the role of interpreters in investigative interviews. *Training, Language and Culture*, *3*(1), 85-104. <https://doi.org/10.29366/2019tlc.3.1.6> |
| Powell, M. B., Manger, B., Dion, J., & Sharman, S. J. (2017). Professionals’ perspectives about the challenges of using interpreters in child sexual abuse interviews. *Psychiatry, psychology and law*, *24*(1), 90-101. https://doi.org/10.1080/13218719.2016.1197815 |
| Splevins, K. A., Cohen, K., Joseph, S., Murray, C., & Bowley, J. (2010). Vicarious post-traumatic growth among interpreters. Qualitative Health Research, 20(12), 1705-1716. https://doi.org/10.1177/1049732310377457 |
